# The Effect of Purified Opharin Isolated from the Venom of King Cobra (*Ophiophagus hannah*) in Modulating Macrophage Inflammatory Responses and Vascular Integrity

**DOI:** 10.3390/toxins16120550

**Published:** 2024-12-19

**Authors:** Tuchakorn Lertwanakarn, Armando Reyes, Emelyn Salazar, Martha Barrientos, Elda E. Sanchez, Montamas Suntravat

**Affiliations:** 1 Department of Physiology, Faculty of Veterinary Medicine, Kasetsart University, Bangkok 10310, Thailand; tuchakorn.l@ku.th; 2 National Natural Toxins Research Center (NNTRC), Texas A&M University-Kingsville, Kingsville, TX 78363, USA; armando.reyes@students.tamuk.edu (A.R.); martha.barrientos@students.tamuk.edu (M.B.); elda.sanchez@tamuk.edu (E.E.S.); 3 Department of Chemistry, Texas A&M University-Kingsville, MSC 161, Kingsville, TX 78363, USA

**Keywords:** king cobra venom, opharin, monocyte-derived macrophages (MDM), inflammatory cytokines, vascular permeability

## Abstract

King cobra (*Ophiophagus hannah*) venom comprises a diverse array of proteins and peptides. However, the roles and properties of these individual components are still not fully understood. Among these, Cysteine-rich secretory proteins (CRiSPs) are recognized but not fully characterized. This study investigates the biological effects of Opharin, the CRiSP from king cobra venom (KCV). The effects of Opharin on cytokine production, specifically on IL-1β, IL-6, IL-8, TNF-α, and IL-10 release, were evaluated over 24 h in monocyte-derived macrophage (MDM) cells. Notably, the levels of these inflammatory cytokines were significantly increased over 24 h, with values higher than those observed in cells treated with crude KCV at most time points. Additionally, the in vivo Miles assay in mice revealed that Opharin increased vascular permeability by 26% compared to the negative control group. These findings highlight the Opharin’s role in severe inflammatory and vascular responses observed in king cobra envenomation. Still, further research is essential to elucidate the pharmacological and toxicological effects of venom components, ultimately enhancing the clinical management of envenomation.

## 1. Introduction

The king cobra (*Ophiophagus hannah*) is one of the most common venomous snakes in Asia, which has been classified as of secondary medical importance, according to the World Health Organization [1]. Envenomed patients commonly exhibited local tissue damage and neurological symptoms, such as paralysis, respiratory failure, or sudden death [2]. Specifically, king cobra venom (KCV) contains a cocktail of proteins and peptides, including neurotoxins, cardiotoxins, phospholipase A_2_ (PLA_2_) enzymes, metalloproteinases, snake venom cysteine-rich secretory proteins (svCRiSPs), and L-amino acid oxidases [3,4,5]. These components produce severe physiological effects upon envenomation [2,6], such as neurotoxins or β-cardiotoxin, which can cause paralysis via post-synaptic inhibition or induce cardiovascular collapse by interfering with cardiac functions [7,8,9,10,11]. Additionally, PLA_2_s disrupt cell membranes, leading to cell lysis and inflammation, while metalloproteinases degrade extracellular matrix components, promoting tissue damage and hemorrhage [12,13]. Despite the extensive characterization of these venom components, the physiological effects of other compounds remain unelucidated. Understanding the specific components of king cobra venom and their toxicological effects is crucial for developing effective treatments and antivenoms [14,15].

Snake venom cysteine-rich secretory proteins (svCRiSPs) are a class of non-enzymatic proteins present in the venoms of various snakes with an estimated molecular mass of 20–30 kDa [16]. The structure of svCRiSP mostly contains five α-helices and eight β-sheets, with 16 conserved cysteine residues [17]. Previously, svCRiSPs have demonstrated their effectiveness in inhibiting L-type calcium, potassium, and cyclic nucleotide-gated ion channels [18,19,20,21]. For example, Wang et al. demonstrated the effect of natrin, the svCRiSP isolated from the venom of *Naja atra*, on the blockade of calcium-activated potassium channels in the mice aortic smooth muscle, resulting in the reduction in vascular tone [22]. Additionally, svCRiSPs from the Southern Pacific rattlesnake (*Crotalus oreganus helleri*), Hellerin, and Mojave rattlesnake (*Crotalus scutulatus scutulatus*) have demonstrated their effects on interfering with vascular permeability and inducing inflammatory responses [23,24,25], which are key contributors to inflammation and vascular leakage [26]. These effects highlighted the mechanisms of the venom ingredient to induce local and systemic inflammation in envenomed patients.

Although the svCRiSP derived from KCV, known as Opharin, has been previously isolated and identified [4], its specific physiological effects remain unknown, particularly its effects on inflammatory responses and vascular permeability. Our study seeks to investigate the impact of the crude KCV and the purified Opharin on cellular inflammatory responses and vascular integrity to provide new insights into the mechanisms by which this protein contributes to the symptoms observed in envenomated patients.

## 2. Results

### 2.1. Isolation and Purification of Opharin from the King Cobra Venom

**Figure 1:**
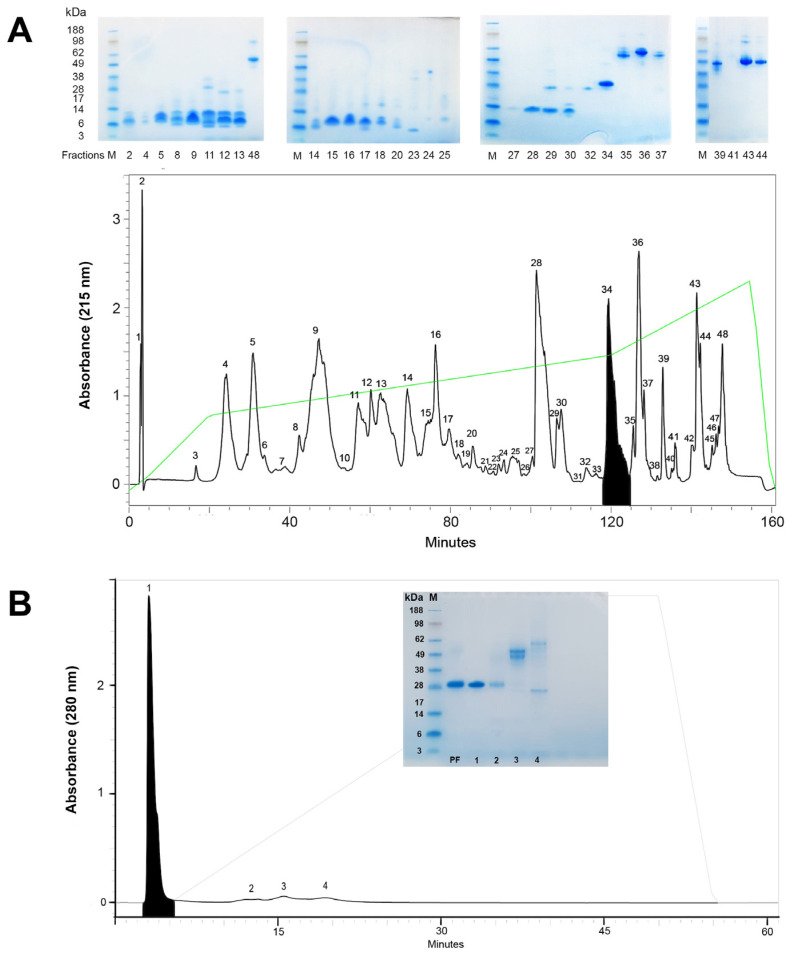
Two-step purification of Opharin from crude KCV. (**A**) An RP-HPLC chromatogram revealed the fractionation of crude venom into 48 distinct fractions. Opharin was specifically detected in the 34th fraction (shaded), with an estimated molecular mass of 28 kDa, as verified by SDS-PAGE (upper panel). The green line demonstrates the gradient of the elution buffer. (**B**) Subsequent fractionation of the pooled 34th fraction (PF) using aIEX chromatography produced four subfractions. Opharin was identified within the first subfraction (shaded) and further confirmed through SDS-PAGE analysis (inset). The gray line represents the gradient of the elution buffer. M = protein marker.

Purification of Opharin from crude KCV was successfully accomplished through a sequential process involving RP-HPLC followed by anion exchange (aIEX) chromatography (Figure 1). During the first step, the crude venom was fractionated into 48 distinct fractions (Figure 1A). The 34th fraction contained a 28 kDa protein with an amino acid sequence that matched against KCV-CRiSP (Opharin), as confirmed by both SDS-PAGE analysis and N-terminal sequencing (Table 1). This fraction was then pooled and subjected to aIEX chromatography. The aIEX chromatogram revealed four subfractions, with the KCV-CRiSP specifically detected in the first subfraction (Figure 1B). As verified by SDS-PAGE and N-terminal sequencing, we obtained the purified KCV-CRiSP (Opharin) from the first subfraction (Table 1). This purified Opharin was subsequently tested for in vitro cell-based assays on endothelial cells and macrophages and in vivo vascular permeability assays.

### 2.2. Cellular Cytotoxicity and In Vitro Endothelial Permeability in Response to Opharin

We assessed cell cytotoxicity in monocyte-derived macrophages (MDM), human dermal lymphatic endothelial cells (HDLEC), and human dermal blood endothelial cells (HDBEC) using the CellTiter-Blue^®^ assay to determine the optimal concentration of the toxin that would not induce cell death or damage. This assay has been used to assess the cytotoxicity of toxins or drugs by measuring cell viability across different cell types [27,28,29], and it has demonstrated reproducibility when compared to other spectroscopic assays [27]. It is important to clarify that the CellTiter-Blue^®^ assay directly measures cell viability while also serving as an indirect indicator of cell cytotoxicity. In our analysis, we interpret decreases in cell viability as indicative of toxic effects. Notably, no evidence of morphological alterations or cell death was exhibited throughout the study period. Additionally, Opharin did not show any effect on the cellular permeability of both HDLECs and HBLECs from the transwell permeability assay.

### 2.3. The Production of Inflammatory Cytokines in Opharin-Treated MDM Cells

**Figure 2:**
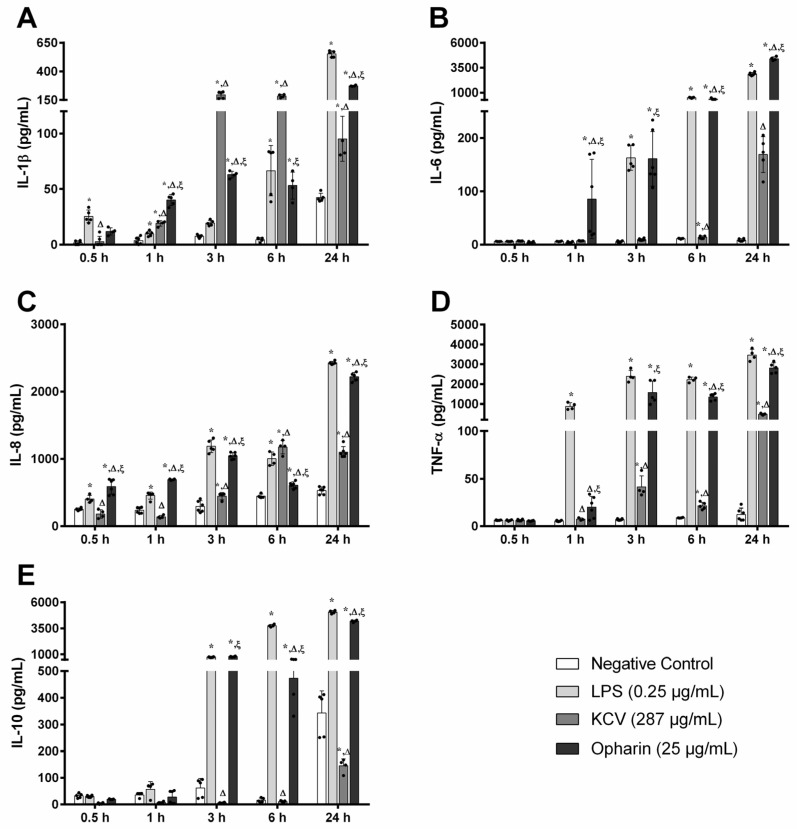
Evaluation of inflammatory cytokine production in MDM cells treated with saline (negative control), lipopolysaccharide (0.25 μg/mL; LPS, positive control), crude KCV (287 μg/mL), and Opharin (25 µg/mL). The release of inflammatory mediators, including (**A**) IL-1β, (**B**) IL-6, (**C**) IL-8, (**D**) TNF-α, and (**E**) IL-10, was quantified at various time points using ELISA. Results are expressed as mean ± SD, n = 4–6 per treatment (black dots). * *p* < 0.05 compared to the negative control, Δ *p* < 0.05 compared to LPS, and ξ *p* < 0.05 compared to KCV.

We further explored the effects of Opharin on cytokine release in MDM cells by measuring the levels of IL-1β, IL-6, IL-8, TNF-α, and IL-10 over a 24 h period (Figure 2). Specifically, the IL-1β level in Opharin-treated cells increased steadily from 0.5 h post-incubation (hpi) (12 ± 3 pg/mL) to 1 hpi (40 ± 5 pg/mL), 3 hpi (63 ± 3 pg/mL), 6 hpi (53 ± 12 pg/mL), and 24 hpi (272 ± 5 pg/mL) (Figure 2A). These findings were consistent with LPS-treated cells where IL-1β levels were significantly increased at 0.5 hpi (25 ± 6 pg/mL), 1 hpi (10 ± 2 pg/mL), 3 hpi (19 ± 2 pg/mL), 6 hpi (67 ± 23 pg/mL), and 24 hpi (552 ± 27 pg/mL). Although Opharin-treated cells produced higher IL-1β levels than other groups at 1 hpi, their levels were significantly lower than the KCV-treated cells at 3 hpi (196 ± 29 pg/mL) and 6 hpi (185 ± 14 pg/mL). However, by 24 hpi, the levels of IL-1β in Opharin-treated cells were significantly higher than those in KCV-treated cells (95 ± 20 pg/mL).

Meanwhile, the production of IL-6 in Opharin-treated cells rose significantly at each time point, from 0.5 hpi (4 ± 2 pg/mL) to 1 hpi (86 ± 74 pg/mL), 3 hpi (161 ± 51 pg/mL), 6 hpi (350 ± 71 pg/mL), and 24 hpi (4408 ± 189 pg/mL) (Figure 2B). Notably, their levels were substantially higher than those in the KCV group at all time points (*p* < 0.05). Additionally, in the LPS-treated cells, IL-6 levels remained unchanged until 1 hpi and were markedly elevated at 3 hpi (163 ± 23 pg/mL), 6 hpi (490 ± 39 pg/mL), and 24 hpi (2866 ± 154 pg/mL), which were significantly higher than the control and KCV-treated cells. Interestingly, cells treated with KCV exhibited an increased production of IL-6 after 24 hpi (178 ± 33 pg/mL).

We also observed significant increments in IL-8 levels in Opharin-treated cells from 0.5 hpi (588 ± 112 pg/mL) to 1 hpi (694 ± 10 pg/mL), 3 hpi (1045 ± 48 pg/mL), 6 hpi (610 ± 50 pg/mL), and 24 hpi (2222 ± 65 pg/mL) (Figure 2C). In comparison, IL-8 levels in LPS-treated cells increased at 0.5 hpi (404 ± 55 pg/mL), 1 hpi (456 ± 54 pg/mL), 3 hpi (1189 ± 94 pg/mL), 6 hpi (1003 ± 101 pg/mL), and 24 hpi (2426 ± 22 pg/mL). Although the IL-8 production in KCV-treated cells reached its peak at 6 hpi (1176 ± 101 pg/mL), their levels were significantly lower than those in the Opharin and LPS groups at other time points.

The release of TNF-α in Opharin-treated cells increased significantly after 3 hpi (1574 ± 576 pg/mL), 6 hpi (1346 ± 173 pg/mL), and 24 hpi (2813 ± 260 pg/mL) (Figure 2D). Additionally, in LPS-treated cells, TNF-α levels remained unchanged at 0.5 hpi, increased at 1 hpi (882 ± 167 pg/mL), 3 hpi (2398 ± 295 pg/mL), 6 hpi (2230 ± 130 pg/mL), and 24 hpi (3469 ± 287 pg/mL). Similarly, an increase was observed in KCV-treated cells from 3 hpi (41 ± 12 pg/mL), 6 hpi (21 ± 4 pg/mL), and 24 hpi (48 ± 38 pg/mL). However, the levels of TNF-α in the groups treated with Opharin and LPS were significantly higher than those produced in the KCV group after 1 hpi (*p* < 0.05).

Finally, IL-10 levels in the Opharin-treated cells markedly increased at 3 hpi (794 ± 36 pg/mL), 6 hpi (473 ± 97 pg/mL), and 24 hpi (4190 ± 105 pg/mL) (Figure 2E). Likewise, in LPS-treated cells, IL-10 levels remained steady until at 1 hpi (57 ± 28 pg/mL) and were markedly elevated at 3 hpi (748 ± 34 pg/mL), 6 hpi (3751 ± 121 pg/mL), and 24 hpi (5062 ± 146 pg/mL). In contrast, IL-10 levels in KCV-treated cells showed a delayed increase, peaking after 24 hpi (145 ± 26 pg/mL), which was significantly lower than the levels in the Opharin group.

### 2.4. In Vivo Vascular Permeability Effects of Opharin

**Figure 3:**
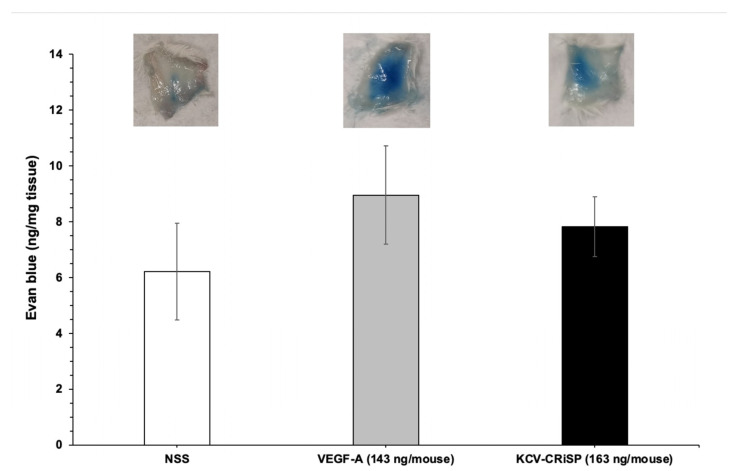
Assessment of in vivo vascular permeability in mice using the Miles assay. Mice received an intravenous injection of 100 μL of Evans blue dye, followed promptly by a subcutaneous injection of normal saline solution (NSS), VEGF-A (143 ng/mouse), or Opharin (163 ng/mouse). Results were assessed from five mice and are expressed as mean ± S.D.

The effect of Opharin on vascular permeability was evaluated using the Miles assay in mice (Figure 3). The concentration of Evan’s blue dye detected in the tissue of the mice injected with 163 ng/mouse of Opharin and 143 ng/mouse of VEGF-A was 7.8 ng/mg tissue and 9 ng/mg tissue, respectively. Despite no statistical significance being observed among groups, the vascular permeability of the mice in the Opharin and VEGF-A groups has increased by 26% and 44%, respectively, compared to the negative control (normal saline solution; NSS).

## 3. Discussion

King cobra envenomation is one of the global health concerns, with cases reported in Southeast Asian countries such as Myanmar [30], Vietnam [31], and the Philippines [32], as well as in the United Kingdom [33] and the United States [34]. Previous studies have identified specific venom components responsible for clinical manifestations. For example, the alpha neurotoxin, α-Elapitoxin-Oh3a, blocks the post-synaptic neuron, causing neuromuscular paralysis [35], while the purified β-cardiotoxin interferes with actomyosin ATPase activity, resulting in suppression of cardiac functions [10]. Nevertheless, the pathogenesis of snakebites with local tissue swelling and generalized inflammation from snakebites remains poorly understood. This study successfully isolated and purified Opharin, the svCRiSP derived from KCV, and revealed its impact on inflammatory cytokine production and vascular permeability.

Our study utilized two-step chromatography to purify Opharin from the crude KCV, which has been effectively reported in the isolation of other svCRiSPs, such as Hellerin from the Southern Pacific Rattlesnake (*C. o. helleri*) and Css-CRiSP from the Mojave Rattlesnake (*C. s. scutulatus*) [23,24,25]. Additionally, the estimated molecular mass of Opharin at 28 kDa was aligned with other svCRiSPs, which confirmed its identity by the first 14 amino acid residues [21,23,24,36]. These findings demonstrated the effectiveness of these techniques in isolating and purifying svCRiSP from other venoms to study their biological activities.

In this study, we tested 25 µg/mL (or 1 µM) of Opharin for the in vitro studies based on previous works with svCRiSPs showing the activation of cultured cells and the expression of molecules related to modulation of the immune response after treatment for up to 24 h with this concentration of toxin [23,24]. Moreover, human MDM cells were used to investigate the in vitro effects of Opharin on the production of inflammatory cytokines. These human macrophages have been widely used as a model to explore the inflammatory responses during viral infection [37,38] and various snake toxins [24,39,40]. It is well-documented that certain snake toxins, such as snake venom metalloproteinases (SVMPs), can activate the inflammatory process through their effects on endothelial cells. This includes upregulating adhesion molecules, facilitating leukocyte adhesion and migration [41], and directly disrupting endothelial integrity, leading to increased vascular permeability [42,43]. However, macrophages play a critical role in the initiation of the inflammatory response, along with other resident cells, by recognizing foreign substances and toxins via pattern recognition receptors (PRRs), such as Toll-like receptors, and releasing mediators that in turn will activate the neighboring cells, including the endothelial cells [44,45,46], or through the activation of inflammasomes, including the NLRP3 complex [47].

Our findings revealed that Opharin significantly induces inflammatory responses in MDM cells, as demonstrated by the elevation of cytokine production such as IL-1β, IL-6, IL-8, TNF-α, and IL-10. In general, IL-1β, IL-6, IL-8, and TNF-α are key mediators of the proinflammatory responses, promoting leukocyte infiltration to the site of inflammation and influencing apoptosis, necrosis, and cell survival pathways [48,49,50,51]. Conversely, IL-10 is an anti-inflammatory cytokine that regulates immune responses by limiting excessive inflammation [52]. Previously, elapid CRiSPs, such as Nk-CRiSP, have been shown to significantly elevate proinflammatory cytokine production, including IL-1β, COX-2, TNF-α, and IL-6, by activating inflammatory responses through the TLR4 signaling pathway in macrophages [53]. In addition, Salazar et al. reported that Css-CRiSP can trigger the innate immune response, recruit leukocytes, and promote local tissue damage and inflammation [24]. Hence, the increased production of these cytokines in Opharin-treated cells may play an important role in the early development of immune responses and local tissue swelling observed in king cobra bite patients [19,35].

Besides assessing the effects of Opharin, we included a group of MDMs treated with whole venom to evaluate the responses and determine the possible contribution of Opharin to the inflammatory effects induced by the whole venom. In this sense, we performed the assays using a concentration of whole venom where we could reflect the concentration of svCRiSP used by itself, but that would also represent the proportion of this toxin in the proteome of king cobra venom (~9%) [54,55]. Notably, we observed that Opharin induces the release of cytokines, especially IL-1β and IL-8, after 0.5 hpi, and IL-6 after 1 hpi. These findings suggested that this purified Opharin possesses a greater effect on activating acute proinflammatory response than the crude venom. We speculated that this difference might be due to other components in the crude venom, such as prostaglandins or natriuretic peptides [5]. While prostaglandins are generally known for their role in promoting inflammation, they can also drive anti-inflammatory responses, particularly in macrophages [56]. For instance, prostaglandin E2 has been shown to influence M2 macrophage polarization or anti-inflammatory profile [57]. Additionally, natriuretic peptides are well-known components that could inhibit cytokine production from the macrophages, such as TNF-α and IL-1β [58,59]. Thus, it is crucial to investigate other specific components of KCV that may provide other physiological effects to gain a comprehensive understanding of this snake envenomation.

Our findings revealed that purified Opharin increased vascular permeability by 26% from the control mice within 30 min post-challenge. Likewise, this effect was previously reported in crotaline CRiSPs, which induce acute effects on vascular and endothelial permeability both in vitro and in vivo [23,25]. Nevertheless, Opharin did not affect either blood or lymphatic endothelial cells in vitro, indicating that the vascular leakage observed in the Opharin-treated group may not directly affect the endothelial barrier function. In general, vascular integrity is primarily regulated by endothelial cell adhesion molecules such as junction adhesion molecules, occludin, claudin, and cadherin families [60]. These proteins, along with VE-cadherin and junctional adhesion molecules (JAMs), maintain vascular integrity and are crucial for endothelial barrier function [61]. However, vascular permeability can also be indirectly regulated by proinflammatory cytokines [62] by disrupting tight junction proteins by IL-1, IL-6, TNF-α, and IL-8 [63,64,65]. Since we observed a significant release of IL-1β and IL-8 from macrophages in vitro at 0.5 hpi, we, therefore, proposed that the mechanism of Opharin-inducing vascular leakage may be related to the activation and recruitment of inflammatory cells, with increased production of proinflammatory cytokines that could potentially lead to increased vascular permeability [66,67]. Previously, chemokines such as fibroblast growth factors [68], CAF-secreted chemokines [69], vascular endothelial growth factors [70], or neutrophil elastase [71] that were released from various cell types at the local skin tissue were associated with the vascular leakage. Therefore, future investigations of the direct effects of Opharin on keratinocytes, fibroblasts, endothelial cells, and other leukocytes such as neutrophils should be performed to assess its effects on skin inflammation, wound healing, cell recruitment, and vascular responses. Additionally, further research is required to investigate the effects of Opharin at different dosages and time intervals on local and systemic inflammatory responses in vivo. This research could yield valuable insights into the pathology of king cobra envenomation, particularly the mechanisms underlying local tissue effects and generalized inflammation.

Taken together, it is suggested that the local tissue swelling or necrosis seen in patients envenomed by KCV may be related to the effect of Opharin that promotes inflammatory processes that lead to increased vascular leakage. While our current study focused on inflammatory cytokine production in MDMs to explore the immunomodulatory effects of Opharin, investigating its specific interactions with endothelial cells is indeed an important aspect for future research. We acknowledge the importance of extending this work to endothelial models, such as examining microvesicle formation, toxin binding to the cell surface, and ultrastructural analyses, which would provide a more comprehensive perspective on Opharin’s mode of action and help elucidate subtle cellular changes in membrane dynamics or intracellular signaling pathways. Moreover, further investigations into the in vivo pharmacological and toxicological effects of venom components will help us understand the mechanisms of tissue damage in snakebite victims and provide valuable information for future patient treatment.

## 4. Conclusions

This study on Opharin, a svCRiSP derived from KCV, highlights its inflammatory effects on cytokine production and vascular permeability, indicating its potential role in promoting local tissue damage and inflammation. These findings contribute to a better understanding of snakebite pathophysiology and emphasize the importance of investigating venom components for improved patient treatment strategies. Further research into the pharmacological and toxicological effects of Opharin and other venom components is crucial for elucidating the mechanisms underlying tissue damage in snakebite victims. The purification and biological effects of Opharin, as explored in the document, demonstrate its significant impact on macrophage inflammatory responses and vascular integrity, with in vivo studies showing a substantial increase in vascular permeability. Understanding Opharin’s role in severe inflammatory and vascular responses observed in king cobra envenomation is essential for enhancing the clinical management of envenomation.

## 5. Materials and Methods

### 5.1. Purification of Opharin from the Crude King Cobra Venom

The crude venom of the king cobra was purchased from the Queen Saovabha Memorial Institute, Bangkok, Thailand. The venom was sourced from a stock obtained from ten adult individuals (five males and five females) during routine venom extraction conducted by the institute in October 2016. The purification of Opharin involved a two-step chromatography process: reverse phase HPLC (RP-HPLC) followed by anion-exchange chromatography (aIEX). Initially, lyophilized KCV was reconstituted in 0.1% trifluoroacetic acid (TFA; Thermo Scientific, Rockford, IL, USA) and filtered using an Acrodisc^®^ 0.45 µm GH Polypro membrane (Pall Corporation, Ann Arbor, MI, USA). The filtered venom was then applied to a PROTO 300 C18 column (5 µm, 300 Å, 4.6 mm × 250 mm, Higgins Analytical, Inc., Mountain View, CA, USA). The column was pre-equilibrated with 0.1% *v/v* TFA in water (solution A) and eluted with 80% *v/v* acetonitrile in 0.1% TFA (solution B). The binary HPLC system (Waters^®^, Milford, MA, USA) operated at a flow rate of 1 mL/min with the following gradient profile: 5 min of 5% isocratic solution B, a linear gradient to 40% solution B over 95 min, a further increase to 70% solution B over 20 min, followed by 10 min of isocratic 70% solution B. Proteins were detected at 215 nm using a UV/visible light detector (Waters^®^, Milford, MA, USA), and fractions were collected manually. The fraction containing Opharin, confirmed by SDS-PAGE and N-terminal sequencing, was pooled together, and buffer-exchanged into 0.02 M Tris A, pH 8, using the Amicon^®^ Ultra 3 kDa (Millipore, Carrigtwohill, Ireland) ultrafiltration unit.

For further purification, the ultrafiltrate was applied to the Protein-Pak DEAE 5PW Column (10 µm, 7.5 mm × 75 mm, Waters^®^, Milford, MA, USA), equilibrated with 0.02 M Tris A, pH 8 (solution A’) and eluted with 0.02 M Tris containing 0.5 M NaCl, pH 8 (solution B’). The flow rate was set at 1 mL/min with the following gradient: 5 min of isocratic 0% solution B’, a linear gradient to 70% solution B’ over 30 min, 5 min of isocratic 70% solution B’, and a linear gradient to 100% solution B’ over 5 min. Opharin was confirmed from each collected fraction using SDS-PAGE and N-terminal sequencing. Finally, solutions were desalted and concentrated using the Amicon^®^ Ultra^®^ 3 kDa centrifugal filter unit (Millipore, Carrigtwohill, Ireland), and the proteins were kept in sterile phosphate-buffered solution (PBS) and stored at −80 °C until use.

We determined the endotoxin content of Opharin using the Pierce™ Chromogenic Endotoxin Quant Kit (Thermo Scientific, Rockford, IL, USA). Briefly, a 96-well plate was pre-warmed at 37 °C, followed by adding 50 µL of standard or Opharin. Fifty microliters of amebocyte lysate were added to each well and incubated for 26 min at 37 °C. After this, 100 µL of chromogenic substrate was added and incubated for 6 min at 37 °C. The reaction was stopped with a 25% acetic acid solution and immediately read at 405 nm in a MultiSkan High microplate reader (Thermo Scientific, Rockford, IL, USA). The sample showed a value of 0.4 endotoxin unit (EU)/mL, which was within the acceptable threshold of 1 EU/mL.

### 5.2. SDS-PAGE and Automated N-Terminal Sequencing

Protein profiles from each fraction from HPLC were separated in a precast 4–12% Bis-Tris SDS-PAGE gel (NuPAGE^®^, Novex, Invitrogen, Carlsbad, CA, USA) and run at 100 V for 95 min using a PowerPac Basic system (Bio-Rad, Richmond, CA, USA) under the reducing conditions using β-mercaptoethanol as a reducing agent. Following electrophoresis, gels were stained with SimplyBlue™ SafeStain (Life Technologies, Carlsbad, CA, USA). For N-terminal sequencing, the fractions were transferred from an SDS-PAGE gel onto a 0.45 µM polyvinylidene fluoride membrane (Immobilon™-P, Millipore, Carrigtwohill, Ireland) using a semi-dry transblot cell system (Bio-Rad, Richmond, CA, USA) at 25 V for 1.5 h. The resulting bands were manually excised and subjected to an automated Edman degradation N-terminal sequencer (PPSQ-33B; Shimadzu, Kyoto, Japan), and the first 14 amino acid residues were determined (PPSQ-30 Analysis; Shimadzu, Kyoto, Japan). The obtained sequences were searched against an online database (https://blast.ncbi.nlm.nih.gov/Blast.cgi?PAGE=Proteins&) accessed on 25 December 2016, to identify the protein. Fractions with the confirmation of Opharin were selected for further in vitro and in vivo studies.

### 5.3. Cell Lines and Culture Conditions

Human monocyte-derived macrophages (MDMs) used to assess cellular responses to Opharin in this study were differentiated from the human monocytic U-937 cell line (ATCC^®^ CRL1593.2™, Manassas, VA, USA) using the protocol described elsewhere [39,72]. Cells were cultured in 75 cm^2^ flasks under constant conditions (5% CO_2_, 37 °C) and maintained with RPMI-1640 medium (ATCC^®^, Manassas, VA, USA), supplemented with 10% fetal bovine serum (FBS), 50 U/mL penicillin, and 50 μg/mg streptomycin. After reaching confluency, cells were seeded into 6-well plates at a density of 3 × 10^6^ cells/well. Then, cells were incubated for 24 h with 20 nM of phorbol 12-myristate 13-acetate (PMA, Sigma-Aldrich, Saint Louise, MO, USA) to induce differentiation into a macrophagic phenotype, specifically monocyte-derived macrophages (MDMs). Following this induction, cells were allowed to rest in RPMI-1640 containing 2% FBS for 48 h before the studies [36].

Human dermal blood endothelial cells (HDBECs) and human dermal lymphatic endothelial cells (HDLECs) were purchased from PromoCell (PromoCell GmbH, Heidelberg, Germany). The cells were cultivated in gelatin-coated 75 cm^2^ flasks at 37 °C with 5% CO_2_ under constant humidity. HDBECs were nurtured with endothelial cell growth media MV, while HDLECs were nourished with MV2 (PromoCell GmbH, Heidelberg, Germany), both supplemented with 5% FBS following the manufacturer’s instructions. All experiments were conducted in passages 3–6 to ensure consistent data.

### 5.4. Cell Cytotoxicity Assay

The cell cytotoxicity assessment was performed in accordance with previously established protocols [24]. MDM, HDBEC, and HDLEC were seeded in a 96-well assay plate at 2 × 10^5^ cells/well and cultured overnight in their corresponding cell media. The following day, the cells were treated with 10 µL of Opharin (0.4–50 µg/mL), crude KCV (0.02–2.6 mg/mL), PBS (negative control), or 1% Triton X-100 for 24 h at 37 °C in 5% CO_2_. After this, cells were then incubated with 20 µL of CellTiter-Blue^®^ reagent (Promega, Madison, WI, USA) for 3 h at 37 °C. Fluorescence was recorded at 540 nm excitation and 590 nm emission wavelengths using a Fluoroskan Ascent FL (Thermo Scientific, Vantaa, Finland). Cells treated only with PBS were used as a negative control, whereas cells treated with 1% Triton X-100 were considered a positive control for toxicity comparison. The percentage of cell viability was calculated relative to the negative control, which was defined as 100% viability.

The negative control was cells treated with PBS, which was the solution employed to dissolve the toxin and venom; however, in both cases, the samples were further diluted to the final concentrations in cell media.

### 5.5. Endothelial Permeability Assay

We assessed endothelial permeability following the protocol previously described [23,25]. Briefly, HDLECs or HDBECs were cultured until they formed a confluent monolayer on gelatin-coated transwell inserts (Corning, Kennebunk, ME, USA). Cells were treated with 1 µM of Opharin for 1 h at 37 °C. After this, 10 µL of a 10 mg/mL FITC-BSA (Sigma-Aldrich, Saint Louise, MO, USA) was added into the upper compartment and incubated for 30 min at 37 °C. Then, a 100 µL sample was collected from the lower compartment to measure FITC-BSA concentration. Measurement was conducted using a Fluoroskan Ascent FL (Thermo Scientific, Vantaa, Finland) with 485 nm excitation and 538 nm emission. The data are presented as relative fold changes compared to the control group.

### 5.6. Cell Activation Assays

Four groups of MDM cells were seeded in a 6-well plate at 3 × 10^6^ cells/well and cultured overnight in their corresponding cell media. The following day, the cells were treated differently with Opharin (25 µg/mL), crude KCV (287 μg/mL), and lipopolysaccharide (LPS) (0.25 μg/mL) as a positive control and PBS as a negative control. Supernatants were collected at 0.5, 1, 3, 6, and 24 hours post-incubation (hpi) and stored at −80 °C for subsequent analysis to evaluate the release of inflammatory mediators.

### 5.7. Quantification of Inflammatory Mediators

Enzyme-linked immunosorbent assay (ELISA) commercial kits were used to assess the levels of inflammatory mediators, including IL-1β (R&D Systems, Minneapolis, MN, USA), IL-8 (Peprotech, Cranbury, NJ, USA), IL-6, TNF-α, and IL-10 (Invitrogen, Vienna, Austria) from the samples. The tests were conducted in duplicates and evaluated at various dilutions, determined individually to align with the ELISA detection range provided by each kit. Absorbances were read at 405 or 450 nm according to the manufacturer’s protocols, and concentrations were calculated using a standard curve with recombinant cytokines and expressed as pg/mL.

### 5.8. Miles Assay

Vascular permeability in vivo was assessed in accordance with our previously established protocol [25]. In brief, BALB/c mice (n = 5 per group, 18–21 g) were injected with 100 μL of 1% Evans blue dye in PBS via the tail vein. Immediately following, 100 μL of either saline, vascular endothelial growth factor A (VEGF-A, 143 ng/mouse), or Opharin (163 ng/mouse) was injected subcutaneously into the dorsal region. After 30 min, the mice were sacrificed, and the injection sites were photographed, excised, and collected in 2 mL tubes. Skin samples were weighed and incubated with 1 mL of formamide at 55 °C for 48 h to extract the dye. The samples were then centrifuged at 12,000 rpm for 40 min, and the absorbance of the supernatant was measured at 620 nm. The amount of Evans blue extravasated per mg of tissue was calculated using a standard curve. Saline served as a negative control, while VEGF-A was the positive control. All animal studies adhered to institutional guidelines and were approved by the Institutional Animal Care and Use Committee, National Natural Toxins Research Center, Texas A&M University-Kingsville (IACUC#: 29 November 2021).

### 5.9. Data Analysis

All statistical analyses were performed using GraphPad Prism software 8.0 (GraphPad, San Diego, CA, USA). Differences in inflammatory mediator production by MDMs across experimental groups at various time points were assessed using two-way ANOVA followed by Tukey’s post hoc test. For the in vivo vascular permeability assay, group comparisons were made using one-way ANOVA with Tukey’s post hoc test. A *p*-value of less than 0.05 was considered statistically significant for all experiments.

## Figures and Tables

**Table 1:** N-terminal sequencing results displayed the estimated molecular weight mass and the first 14 amino acid residues of Opharin found from the purification.

Purification Process	Peak	Apparent Mass (kDa)	Amino Acid Sequences	Identification	Accession No.
RP-HPLC	F34	28	GVDFNSEMTRRDKK	Opharin	Q7ZT98.1
aIEx	F1	28	NVDFNSETTRRQKK	Opharin	Q7ZT98.1

## Data Availability

The original contributions presented in the study are included in this article, further inquiries can be directed to the corresponding author.
